# First person – Roberta Azzarelli

**DOI:** 10.1242/bio.058609

**Published:** 2021-02-22

## Abstract

First Person is a series of interviews with the first authors of a selection of papers published in Biology Open, helping early-career researchers promote themselves alongside their papers. Roberta Azzarelli is first author on ‘Three-dimensional model of glioblastoma by co-culturing tumor stem cells with human brain organoids’, published in BiO. Roberta conducted the research described in this article while a Rita Levi Montalcini fellow in Roberta Azzarelli's lab at Unit of Cell and Developmental Biology, Department of Biology, University of Pisa, Italy. She is now a research associate in the lab of Anna Philpott at the Wellcome-MRC Cambridge Stem Cell Institute, University of Cambridge, UK, investigating how stem cell and developmental biology can help tackle cancer.


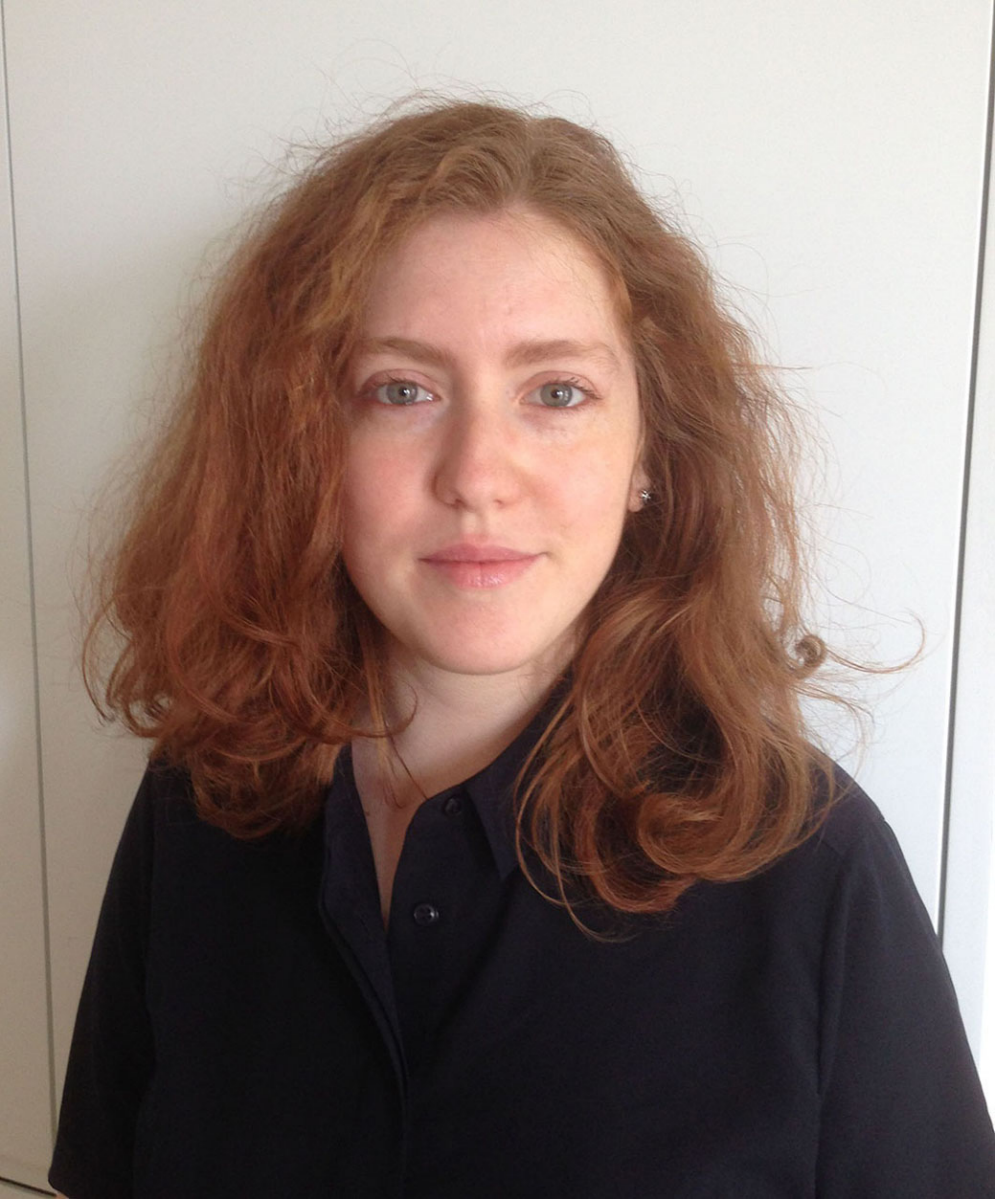


**Roberta Azzarelli**

**What is your scientific background and the general focus of your lab?**

I studied Biotechnology in Milan and did my PhD with Francois Guillemot at the National Institute for Medical Research in London, where I investigated the mechanisms of mammalian brain development. I loved everything about working with embryos, cultures and stem cells. Since then, I have extended my interests into the dynamics of stem cell behaviour in health and cancer. I have studied the function and regulation of proneural transcription factors with Anna Philpott and Ben Simons at the University of Cambridge and moved to cancer biology with an independent fellowship. Currently, I am working on different projects aiming to establish how cells make fate decisions, how they remain faithful to those decisions and understand why they might fail, like in certain types of cancer.

**How would you explain the main findings of your paper to non-scientific family and friends?**

Brain cancer is inevitably hard to study in a human being, especially when it comes to its cellular and molecular details; it is thus vital to have a model that recreates the cancer and its microenvironment in a lab Petri dish. In this paper, we used cells derived from an aggressive brain cancer, called glioblastoma, and provided them with a brain-like environment, so that they could behave like they would in the patient brain. In this system, we can study how tumor cells behave and understand why different tumors behave differently. This can inform the development of personalized therapies in the future.

**What, in your opinion, are some of the greatest achievements in your field and how has this influenced your research?**

In my view, one of the most influential discoveries in the field is somatic cell reprograming. It was during my PhD when Shinya Yamanaka and John Gurdon were awarded the Nobel prize for the discovery that mature somatic cells could be reprogrammed to become pluripotent. The root of this discovery lies in the quest to understand cell specialization and to challenge the previously established assumption that different cell types have different genomes. This discovery influenced our work and most of what we do today in developmental and stem cell biology in various ways. Not only does it provide us with new tools like induced pluripotent stem cells (iPSCs), but it has also fostered new ideas through the conceptual implications of somatic cell fate reprogramming and plasticity.

**What changes do you think could improve the professional lives of early-career scientists?**

If we exclude the only simple answer (more long-term/permanent research jobs, more faculty positions!), then we open a Pandora's box. I believe that the support from your mentor/s is key to improve your professional life, whatever stage you are and I am happy to see more and more institutions taking steps to establish formal mentorship programs. Sometimes these programs are aimed only at independent fellows, but I think they should be extended to anyone who feels the need, from PhDs to postdocs. I also think that early career scientists would benefit from a better structured definition of their career path. It is important to set career expectations, so that the day-to-day fun of carrying out research projects won't be obfuscated by the uncertainty of the future. More involvement of early career scientists in the publishing and funding ‘machines’ will also be helpful. The Company of Biologists created the Node network, a useful database where everyone can register their profile and expertise and that can be used to contact early career scientists for talks, reviews and collaborations, and I think more initiatives like this should be embraced. Finally, I'd like to praise Biology Open for featuring ‘First Person’ interviews in their journal. This is a great way to foster early career scientists!

**Figure:**
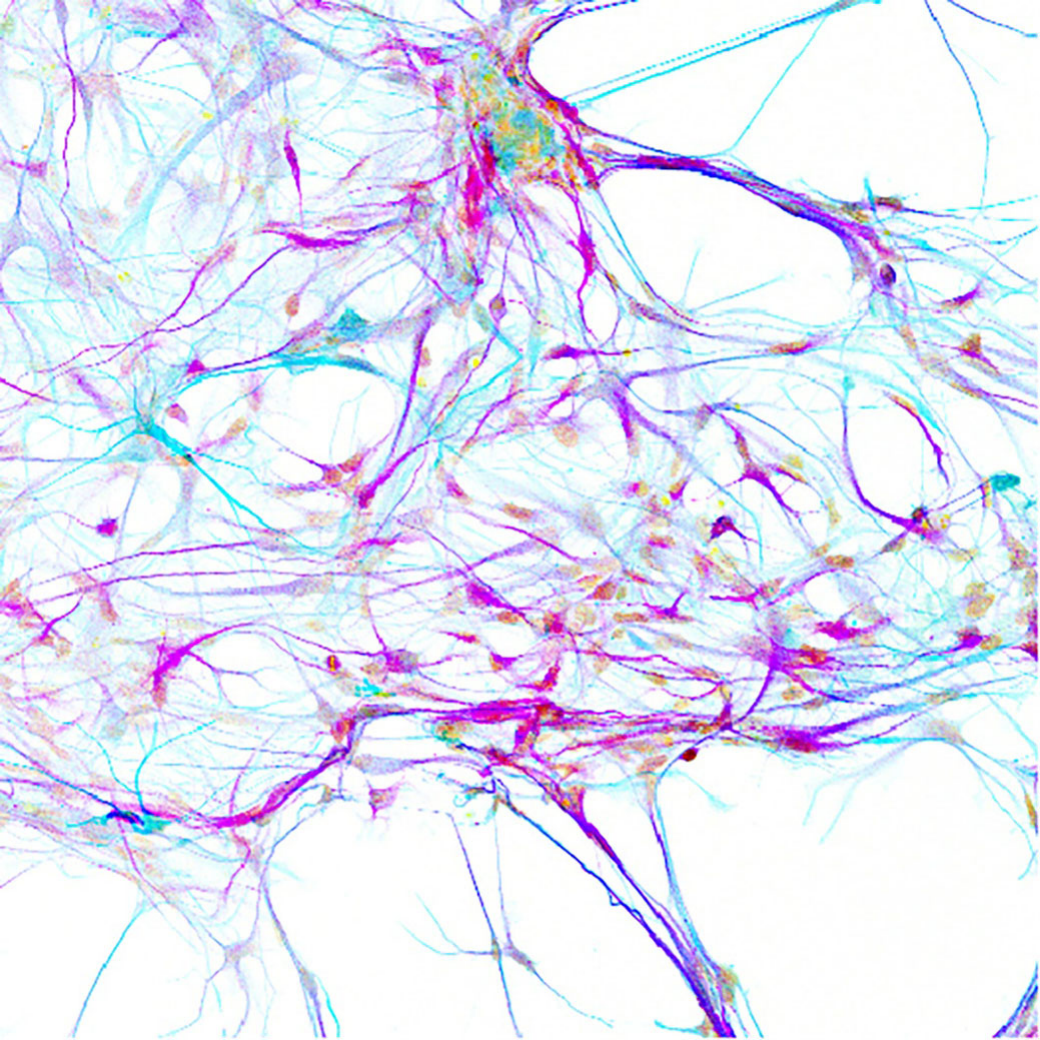
**Brain cancer cells differentiating into neurons (light blue) and glial cells (purple) *in vitro* (shown as inverted colours).**

**What's next for you?**

I am currently setting up exciting projects on cell fate acquisition and maintenance in embryonic stem cells in the lab of Anna Philpott. I am very excited to carry out these projects alongside some more independent work on cancer cells, as I believe similar mechanisms might underlie cell fate fidelity in the two contexts. I am looking forward to being able to combine novel stem cell and organoid technologies with single cell genomic and bioinformatic approaches to get the resolution in fate decisions we have been missing for a long time.
